# Identification and functional analysis of a novel *TRAPPC2* intronic variant in a four-generation Chinese pedigree with SEDT

**DOI:** 10.3389/fgene.2026.1763609

**Published:** 2026-02-09

**Authors:** Yongfen Lyu, Wuhen Xu, Bin Xu, Xiaojun Tang, Man Xiao, Xiaoping Lan, Yongchen Yang, Xiaozhen Song, Shengnan Wu

**Affiliations:** 1 Department of Endocrinology, Shanghai Children’s Hospital, School of Medicine, Shanghai Jiao Tong University, Shanghai, China; 2 Department of Clinical Laboratory, Shanghai Children’s Hospital, School of Medicine, Shanghai Jiao Tong University, Shanghai, China; 3 Institute of Pediatric Infection, Immunity, and Critical Care Medicine, School of Medicine, Shanghai Jiao Tong University, Shanghai, China; 4 Faculty of Medical Laboratory Science, College of Health Science and Technology, School of Medicine, Shanghai Jiao Tong University, Shanghai, China; 5 Department of General Surgery, Shanghai Pudong New Area People’s Hospital, Shanghai, China

**Keywords:** noncanonical splicing variant, short stature, skeletal dysplasia, TRAPPC2, X-linked spondyloepiphyseal dysplasia tarda

## Abstract

**Background:**

Pathogenic variants in the trafficking protein particle complex subunit 2 (*TRAPPC2*) gene are known to cause X-linked spondyloepiphyseal dysplasia tarda (X-linked SEDT), a rare hereditary cause of childhood short stature. Genetic diagnosis is critical in early diagnosis and management of the disease. Majority of the pathogenic variants are predicted to cause premature truncation. However, few have been functionally studied. In this study, we reported a Chinese pedigree with multiple affected remarkably short stature males and described the novel variant by *in-vitro* functional study.

**Methods:**

To complete precise molecular diagnosis and subsequent genetic counseling of a large Chinese pedigree with remarkably short stature. Trio whole exome sequencing was performed on the proband and his parents and phenotype-driven data analysis was conducted. The potentially pathogenic variant was verified by Sanger sequencing in parent-offspring trio and other family members. *In vitro* experiments involving minigene splicing study and protein expression assay were performed for the potentially disease-causing noncanonical splice variant.

**Results:**

Using whole exome sequencing, we identified a novel intronic variant, c.94–11C>G, located in intron three of the *TRAPPC2* gene. Minigene analysis confirmed that this variant resulted in abnormal splicing, leading to a frameshift insertion of 10 nucleotides and a subsequent loss of TRAPPC2 protein expression. This variant has never been reported.

**Conclusion:**

Our findings highlight the pathogenic nature of a novel noncanonical splicing variant, thus expanding the genetic spectrum of *TRAPPC2*-related disorder. Furthermore, this discovery has important implications for genetic counseling, prenatal genetic diagnosis, and follow-up care for affected individuals in this family.

## Introduction

X-linked spondyloepiphyseal dysplasia tarda (X-linked SEDT, OMIM#313400) is a rare, late-onset, progressive skeletal dysplasia, primarily affecting males, leading to childhood disproportionate short stature, defective structures of spinal vertebral bodies and epiphyses of the long bones, and premature degenerative osteoarthritis (Gedeon et al., 2001; Gedeon et al., 1999; Whyte et al., 1999; Fiedler et al., 2004). Characteristic radiographic features include platyspondyly with a superior and inferior hump-shaped deformity of the central and posterior portions, narrow intervertebral disc spaces, flat femoral heads, and short femoral neck (Gedeon et al., 1999; Tiller et al., 2001; Wang et al., 2013; Won et al., 2019; Zhang et al., 2020; Lou et al., 2023; He et al., 2025). X-linked SEDT is linked to pathogenic variants in the *TRAPPC2* gene (OMIM#300202), which is located at chromosome Xp22. *TRAPPC2* contains six exons, with the coding region on exons three to six, encoding a 140-amino acid protein involved in endoplasmic reticulum (ER)-to-Golgi vesicular transport (Gedeon et al., 1999; Zappa et al., 2024). Genetic analysis is required for the confirmatory early diagnosis. To date, 68 different *TRAPPC2* variants have been reported (Human Gene Mutation Database, HGMD 2025.1; http://www.hgmd.cf.ac.uk/ac/index.php), including seven missense, 11 nonsense, 13 splicing, 22 small deletions, two small insertions, one small indels and 12 gross deletions. Majority of the variants are predicted to cause premature truncation. However, few have been functionally studied.

In our study, we identified a novel noncanonical splice-acceptor variant, c.94–11 C>G, in the intron three of *TRAPPC2*, within a large four-generation Chinese pedigree with four affected male family members. Using a minigene assay, we confirmed the c.94–11 C>G variant caused aberrant *TRAPPC2* splicing, resulting in decreased TRAPPC2 expression. This finding expands the *TRAPPC2* genetic variant spectrum, enhances diagnostic confidence for affected individuals in this Chinese family, and provides support for genetic counseling.

## Materials and methods

### Human subjects

**FIGURE 1:**
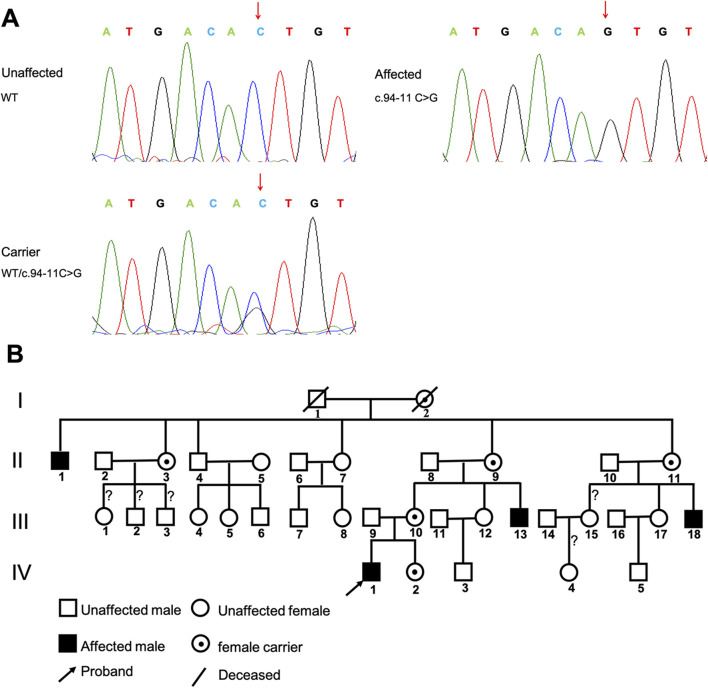
Sequence analysis and pedigree of the Chinese X-linked SEDT family. **(A)** Sequence of unaffected members, affected members and carriers from this family. **(B)** Pedigree of the family. The arrow points to the proband (IV-1). (?) indicated that the genotype had not been obtained due to unavailability of blood samples. Open boxes represent healthy males, open circles represent healthy females, and black boxes represent affected males. Boxes or circles with a crossing line signify deceased individuals. Circles with a dot in the middle indicate carrier status.

The pedigree of the Chinese SEDT family is shown in Figure 1, demonstrating a characteristic X-linked recessive inheritance pattern. The proband, a 16-year-old Chinese male, sought evaluation for short stature at Shanghai Children’s Hospital. Peripheral blood samples and clinical information were collected for available family members.

### Genetics analysis

To elucidate the genetic cause of the disorder, we performed trio whole-exome sequencing (TWES) on the proband and his parents as previously described (Xiaozhen et al., 2021). With participant consent, we extracted genomic DNA for TWES and subsequent Sanger sequencing from peripheral blood samples, employing the DNA Blood Mini Kit (QIAGEN, Germany). An IDTxGen® Exome Research Panel (IDT, USA) was used to capture the exons, and HiseqX10 (Illumina, USA) was used to sequence the DNA fragments. The acquired sequencing data were further annotated and filtered. Minor allele frequency (MAF) < 1% in the gnomAD (https://gnomad.broadin-stitute.org/), pathogenicity prediction algorithms, disease-association database (ClinVar (https://www.ncbi.nlm.nih.gov/clinvar/) and HGMD (http://www.hgmd.cf.ac.uk/ac/index.php)), and disease phenotypes were used to search for all possible disease-cause variants. The potentially pathogenic variants were verified through Sanger sequencing. The pathogenicity of the identified variations was determined following the guidelines set forth by American College of Medical Genetics and genomics and association for molecular pathology (ACMG/AMP) (Richards et al., 2015).

### Functional study of the putative splicing variant in *TRAPPC2*

To validate the effect of c.94–11C > G variation on function, we performed *in vitro* experiments involving a minigene splicing study and protein expression assay. Amplicons containing either wild type or variant allele [intron 3 (211bp)-Exon 4 (145 bp)] were generated by standard PCR and cloned into pcMINI-C vector. We verified the successful construction of the expression vectors through bacterial liquid PCR and Sanger sequencing.

The recombinant vectors (pcMINI-C-TRAPPC2-wt and pcMINI-C-TRAPPC2-mut) were then transiently transfected into human embryonic kidney cells (HEK-293T) and human cervical cancer cells (HeLa), according to the provided instructions. After 48 h of cell culture, total RNA was extracted from both HEK-293T and HeLa cells using the TRIzol method (Thermo Fisher Scientific, Waltham, MA, USA). This RNA was then reverse transcribed into cDNA using a reverse transcription kit (TransGen Biotech, Beijing, China), following the manufacturer’s instructions. Subsequently, standard PCR was performed with primers flanking the minigene. The PCR products were identified by 2% agarose gel electrophoresis and verified by Sanger sequencing. The used primers are listed in Supplementary Table S1.

We synthesized human *TRAPPC2* cDNA (transcript NM_001011658.4), followed by amplification to obtain PHAGE-TRAPPC2-wt (using primers PHAGE-TRAPPC2-SalI-F and PHAGE-TRAPPC2-NotI-R) and pEGFP-TRAPPC2-wt (using primers pEGFP-C1-TRAPPC2-HindIII-F and pEGFP-C1-TRAPPC2-BamHI-R) fragments. Mutant fragments were obtained with TRAPPC2-MUT-F and TRAFPPC2-MUT-R primers. SalI and NotI were used to construct wt or mutant *TRAPPC2* onto the PHAGE vector, and HindIII and BamHI were used for construction onto the pEGFP-C1 vector. We assessed the successful construction of eukaryotic expression plasmids through PCR and Sanger sequencing.

Then we transfected HEK-293T cells with the different expression plasmids 1 μg when the cell density reached 60%. After 48 h, total RNA was isolated from cultured cells and 1 μg of RNA was reverse transcribed to cDNA. The expression of wild type and mutant *TRAPPC2* was detected by qPCR. Total protein was extracted using a Protein Extraction Kit (Sangon Biotech, Shanghai, China). Subsequently, 25 μg of total protein was separated using 8% sodium dodecyl sulfate polyacrylamide gels (SDS-PAGE) and then transferred to nitrocellulose membranes (Merck Millipore, Darmstadt, Germany). After blocking with 5% skim milk, the membranes were incubated overnight at 4 °C with specific primary antibodies. Finally, the bands were detected using ECL Prime Western blotting Reagent (GE Healthcare, Buckinghamshire, UK). The primary antibodies used were rabbit anti-GAPDH (Cell signaling technology; 1:1000), mouse anti-HA (Dia-an; 1:2000), and mouse anti-GFP (Dia-an; 1:1000).

## Results

### Clinical features of the patients with SEDT

**FIGURE 2:**
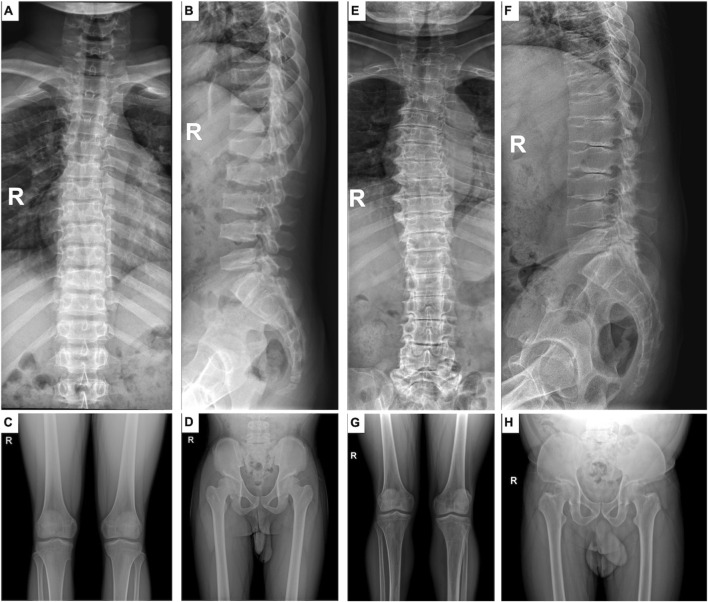
Features of radiograph with SEDL. **(A–D)** radiographs were taken at IV-1 16 years old. **(E–H)** radiographs were taken at III-13 32 years old. **(A,E)** Frontal view showing mild kyphoscoliosis. **(B,F)** Lateral view of the lumbosacral spine, revealing platyspondyly, hump-shaped central and posterior portions of vertebral bodies, and narrow disc spaces. **(C,G)** Frontal view of the knees, displaying mild flattened tibial plateaus. **(D,H)** Frontal view of the pelvis, indicating flat femoral heads and short femoral necks.

The male proband (IV-1) (Figure 1), a 16-year-old Chinese boy, first presented to our clinic with a concern of short stature. He measured 137 cm in height, with an arm span of 150 cm, displaying disproportionate short stature, a short neck, and a barrel-shaped chest. He did not exhibit any other systemic complications. The frontal and lateral radiograph of the spine showed mild kyphoscoliosis (Figures 2A,B). The lateral radiograph of the lumbosacral spine showed platyspondyly and a hump-shaped appearance in the central and posterior portions of vertebral endplates, with no significant narrowing of disc spaces (Figure 2B). Knee radiographs showed flattened tibial plateaus (Figure 2C). Mild flattened femoral heads, coxa vara, irregular hip-joint surfaces and short femoral neck were observed in the radiographs of the pelvis (Figure 2D). The pedigree of the SEDT family was shown in Figure 1. In the family, three other living adult males (II-1, III-13, III-18) experienced similar symptoms and manifestations. Their heights measured 134, 138, and 134 cm, respectively, and they also exhibited disproportionate short stature, short neck, a barrel-shaped chest. Their arm spans exceed their height measurements by 13–14 cm. Case II-1, who was 65 years old, reported severe back, knee and hip pain since the age of 17, with limited knee joint motion. Case III-13, the maternal uncle of the proband, was 32 years old. X-ray radiographs of III-13 showed more typical characteristics of SEDT, including flattening of the vertebral bodies, significantly narrow intervertebral disc spaces, end plate sclerosis, narrow hip-joint surfaces and short femoral necks (Figures 2E–H). Detailed clinical and radiographic findings of the affected males are summarized in Table 1. There were no affected women in the family (the average height was 156.4 cm). The pattern of inheritance supported an X-linked recessive disorder through four generations.

**TABLE 1:** Summary of clinical findings in male individuals with *TRAPPC2* c.94–11C>G variant.

Characteristics	II-1	III-13	III-18	IV-1
General characteristics				
Age at last evaluation (year)	65	32	25	16
Gender	Male	Male	Male	Male
Height in cm	134	138	134	137
Arm span in cm	148	152	147	150
Head circumference in cm	59	60	57	56
Corneal opacities	−	−	−	−
Short stature	+	+	+	+
Short neck	+	+	+	+
Short trunk	+	+	+	+
Barrel-shaped chest	+	+	−	+
Osteoarthritis	+	−	−	−
Limited joint motion	+	−	−	−
Radiological examination				
Kyphoscoliosis	NA	+	NA	+
Platyspondyly	NA	+	NA	+
Hump-shaped mound of bone in central and posterior portions	NA	+	NA	+
Narrow disc spaces	NA	+	NA	−
Coxa vara	NA	+	NA	+
Short femoral neck	NA	+	NA	+
Mild epiphyseal irregularities	NA	+	NA	+
Treatment				
Growth hormone	−	−	−	−
Pain management	+	−	−	−
Replacement of joint	−	−	−	−

NA: not available.

### Genetic finding

TWES and Sanger sequencing revealed a novel hemizygous variant, c.94–11C>G, situated in intron three of the *TRAPPC2* gene (NM_001011658.4), which was inherited from the proband’s mother (Figure 1). Notably, this variant is novel and absent from both the HGMD and the gnomAD. Further Sanger sequencing analysis of this family showed that the same variant was detected as hemizygous in affected individuals (II-1, III-13 and III-18), and multiple maternal female relatives of the proband (II-3, II-9, II-11and IV-2) were carriers of the variant (Figure 1). The remaining males with a normal phenotype tested were negative for this variant. *In silico* tools, Varseak (https://varseak.bio) and SpliceAI (https://github.com/Illumina/SpliceAI), predicted that the c.94–11C>G variant affects normal splicing and generates an aberrant splicing site. No other likely pathogenic or pathogenic (LP/P) variants associated with short stature were found.

### Minigene study of the putative noncanonical splicing variant in *TRAPPC2*

**FIGURE 3:**
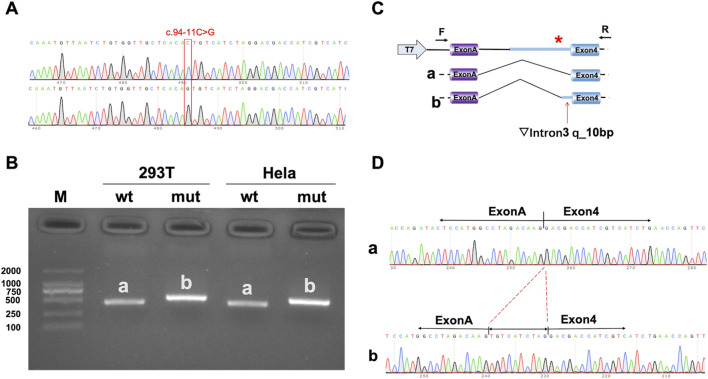
Minigene assay on a noncanonical splice site variant in *TRAPPC2*. **(A)** Sequencing of constructs. **(B)** Separation of reverse-transcription polymerase chain reaction (RT-PCR) products from 293T and Hela cells transfected with either wild-type (wt) or mutant (mut) pcMINI-C vector via electrophoresis. (M: DNA marker) **(C)** Structure of the splicing vector pcMINI-C-minigene *TRAPPC2*, with the symbol “*” representing the variant’s location (top); minigene *TRAPPC2*-wt splicing diagram **(a)**; minigene *TRAPPC2*-mut splicing diagram **(b)**. **(D)** Sequencing of the minigene product showed normal mRNA composing exons A (vector) and exon 4 (*TRAPPC2*) **(a)**, and abnormal mRNA composing 10 bps of intron three retention between exons A (vector) and exon 4 (*TRAPPC2*) **(b)**.

The intronic variant (c.94–11C > G) detected in *TRAPPC2* exhibited a co-segregation feature within this pedigree, indicating its pathogenicity for the disease. Since we could not obtain sufficient blood samples from proband to validate the variation *in vivo*, *in vitro* minigene assays were then performed to determine the effect of the variant on mRNA splicing. Recombinant vector sequencing showed the successful insertion of both wt and mutant *TRAPPC2* minigenes into the pcMINI-C vector (Figure 3A). The minigene assays showed that pcMINI-C-TRAPPC2-wt produced an expected band of approximately 500 bps in both 293T cells and Hela cells (Figure 3B). In contrast, pcMINI-C-TRAPPC2-mut produced a larger band than pcMINI-C-TRAPPC2-wt (Figure 3B), suggesting that c.94–11C > G variant may have caused the abnormal splicing shown in Figure 3C. Sequencing and online alignment of the PCR product showed the retention of an additional 10 bps in intron three of *TRAPPC2* (Figure 3D). These results confirm that the intronic variant c.94–11C > G caused abnormal *TRAPPC2* mRNA splicing, with the retained 10 bps resulted in a frameshift and finally lead to the creation of a premature termination codon (PTC).

### Effect of the abnormal splicing on TRAPPC2 protein expression

Given the abnormal *TRAPPC2* mRNA splicing and the appearance of a PTC, we postulated two potential outcomes: the encoding of a C-terminal truncated TRAPPC2 protein or the activation of nonsense-mediated mRNA decay. To test this hypothesis, we constructed two sets of expression plasmids to verify the effect of aberrant splicing on TRAPPC2 expression.

**FIGURE 4:**
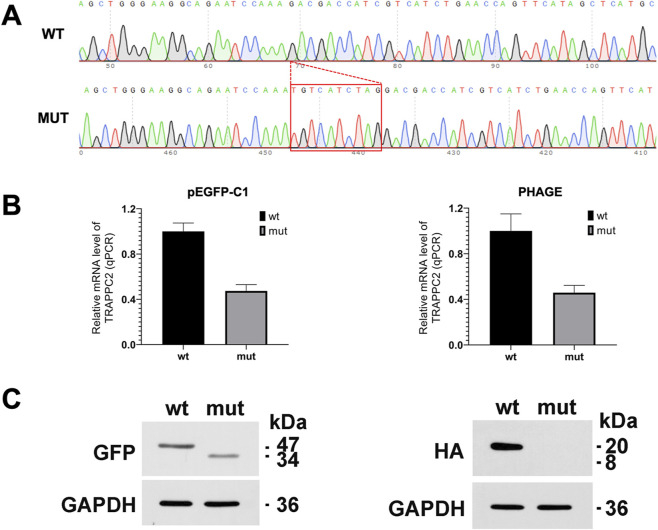
The effect of c.94–11C>G variant on TRAPPC2 expression. **(A)** Expression product sequencing revealed c.94–11C>G led to c.93_94ins tgtcatctag. **(B)** qPCR analysis of mRNA expression. The mRNA expression of the mutant construct was significantly decreased compared to the wt. **(C)** Western blot analysis of protein expression. The protein expression of the mutant construct was significantly lower than that of the wt. wt: wild-type, mut: mutant.

The RT-PCR product sequencing showed that the mut-vector (PHAGE and pEGFP-C1) produced c.93_94ins tgtcatctag (p.Asp32Cysfs*23), indicating that the eukaryotic expression vectors were successfully constructed (Figure 4A). The RT-PCR results also showed that the mRNA expression level of the splicing variant (mut) was indeed significantly lower than those of the wt in both sets of vectors (Figure 4B).

Then we transfected four TRAPPC2 expression constructs into 293T cells to evaluate the effect of variant on protein expression. Finally, we found that the c.94–11C>G variant led to the production of a truncated TRAPPC2 protein with significantly reduced expression compared to the wild type when using the pEGFP-C1 vector. In the case of the PHAGE vector, the mutant construct exhibited no discernible band, indicating that the c.94–11C>G variant activated nonsense-mediated mRNA decay (Figure 4C). These results underscore that the c.94–11C>G variant results in the absence of TRAPPC2 protein, which is required for the export of procollagen from the endoplasmic reticulum to the Golgi apparatus.

## Discussion

X-linked SEDT is a rare but well-defined cause of childhood short stature and skeletal dysplasia. The responsible gene, *TRAPPC2*, was initially reported by Gedeon in 1999 (Gedeon et al., 1999). Here, we report a novel noncanonical splice site variant, c.94–11C>G in *TRAPPC2* gene, in a large Chinese family. Four male patients presented with the typical clinical and radiological features of SEDT. We confirmed this variant as the genetic basis of the condition in this Chinese SEDT family using minigene assay. Our analysis revealed that the c.94–11C>G variant caused the retention of an additional 10 base pairs in *TRAPPC2*, leading to the premature termination of translation and subsequent degradation of the translated protein. As a component of the transport protein particle, the loss of TRAPPC2 may disrupt the ER-to-Golgi vesicle transport process.

With the development of gene sequencing, genetic testing has become an important method for disease diagnosis. Simultaneously, a large number of variants of uncertain significance (VUS) related to the phenotype were detected. Due to difficulties in obtaining patient-derived blood samples or insufficient gene expression in peripheral blood, functional validation of whether these VUS affect pre-mRNA splicing remains challenging. The minigene assay has emerged as a convenient and efficient method to provide functional evidence for variants interpretation and pathogenicity assessment. However, minigene only contain partial exon-intron regions, and cannot simulate the native splicing context of full-length genes. Consequently, the most accurate strategy for splicing validation is to complement with analyses using patient-derived samples whenever feasible.

The *TRAPPC2* gene is composed of six exons with translation start codon in exon3. According to the HGMD, there have been 68 reported *TRAPPC2* variants, most frequently occurring in exons 4-6. These exons are vital for protein binding and maintaining the three-dimensional structure of the TRAPPC2 protein (Choi et al., 2009; Jang et al., 2002). Among these variants, thirteen are splicing mutations, nine of which affect classical splicing sites. The novel variant we discovered in this study, c.94–11C>G, is a noncanonical splicing variant located in intron 3. So far, a total of five pathogenic variants have been detected in intron 3, c.93 + 1G>A, c.93 + 1G>T, c.93 + 5G>C, c.93 + 5G>A and c.94–2A>G, suggesting that intron three is a high-frequency mutation region critical for TRAPPC2’s function. Numerous affected individuals carry splicing variant in intron three from different countries or regions demonstrated phenotypes indistinguishable from those caused by other premature stop codon variants (Tiller et al., 2001; Fukuma et al., 2018; Adachi et al., 2014).

SEDT is a progressive disease that significantly impacts patients' quality of life, and unfortunately, there is no effective treatment (Miyoshi et al., 2004). In this study, patients III-13, III-18 and IV-1 have shown no significant osteoarthritis symptoms to date, presenting with short stature and mild scoliosis. But case II-1, aged 65, reported severe back, knee and hip pain, with limited knee joint motion. Radiographic assessments revealed that IV-1 (16 years old) did not exhibit significant narrowing of disc spaces, while III-13 (32 years old) showed significantly narrowed intervertebral disc spaces and end plate sclerosis. Early diagnosis is helpful in symptomatic treatment. Individuals III-1, III-2, III-3, III-15 and IV-4 declined genetic testing for the c.94–11C>G variant, citing self-reported normal height and absence of other clinical manifestations. These family members will be included in longitudinal follow-up assessment in the future studies.

Unfortunately, antenatal ultrasound cannot diagnose affected fetuses. However, due to the availability of genetic mutation information, genetic counseling, extended family screening, and prenatal diagnosis are all viable options for this family. For carrier females, each pregnancy carries a 50% risk of producing an affected male offspring and a 50% probability of female being a carrier. In contrast, male patients transmit the variant to all daughters, who are obligate carriers, while all sons are unaffected. All carriers-daughters should receive genetic counseling upon reaching adulthood particularly when planning a pregnancy. Preimplantation genetic testing can be considered as a reproductive option.

## Conclusion

In conclusion, by genetic testing and functional studies, we identified a novel pathogenic noncanonical splice variant c.94–11C>G of the *TRAPPC2* gene in this Chinese SEDT family. Our findings expanded the mutation spectrum of the TRAPPC2 gene and enable multiple clinical actions available for this family.

## Data Availability

The original contributions presented in the study are included in the article/Supplementary Material, further inquiries can be directed to the corresponding authors.

## References

[B1] Adachi H. Takahashi I. Takahashi T. (2014). Novel TRAPPC2 mutation in a boy with X-linked spondylo-epiphyseal dysplasia tarda. Pediatr. Int. 56 (6), 925–928. 10.1111/ped.12397 25521980

[B2] Choi M. Y. Chan C. C. Chan D. Luk K. D. Cheah K. S. Tanner J. A. (2009). Biochemical consequences of Sedlin mutations that cause spondyloepiphyseal dysplasia tarda. Biochem. J. 423 (2), 233–242. 10.1042/BJ20090541 19650763

[B3] Fiedler J. Le Merrer M. Mortier G. Heuertz S. Faivre L. Brenner R. E. (2004). X-linked spondyloepiphyseal dysplasia tarda: novel and recurrent mutations in 13 European families. Hum. Mutat. 24 (1), 103. 10.1002/humu.9254 15221797

[B4] Fukuma M. Takagi M. Shimazu T. Imamura H. Yagi H. Nishimura G. (2018). A familial case of spondyloepiphyseal dysplasia tarda caused by a novel splice site mutation in TRAPPC2. Clin. Pediatr. Endocrinol. 27 (3), 193–196. 10.1297/cpe.27.193 30083037 PMC6073055

[B5] Gedeon A. K. Colley A. Jamieson R. Thompson E. M. Rogers J. Sillence D. (1999). Identification of the gene (SEDL) causing X-linked spondyloepiphyseal dysplasia tarda. Nat. Genet. 22 (4), 400–404. 10.1038/11976 10431248

[B6] Gedeon A. K. Tiller G. E. Le Merrer M. Heuertz S. Tranebjaerg L. Chitayat D. (2001). The molecular basis of X-linked spondyloepiphyseal dysplasia tarda. Am. J. Hum. Genet. 68 (6), 1386–1397. 10.1086/320592 11349230 PMC1226125

[B7] He Z. Dai S. M. Chen Z. (2025). Clinical Images: back pain, flattened vertebral bodies, and a novel mutation in the TRAPPC2 gene. ACR Open Rheumatol. 7 (2), e70000. 10.1002/acr2.70000 39948659 PMC11825289

[B8] Jang S. B. Kim Y. G. Cho Y. S. Suh P. G. Kim K. H. Oh B. H. (2002). Crystal structure of SEDL and its implications for a genetic disease spondyloepiphyseal dysplasia tarda. J. Biol. Chem. 277 (51), 49863–49869. 10.1074/jbc.M207436200 12361953

[B9] Lou G. Zhao Y. Zhao H. Zhang Y. Hao B. Qin L. (2023). Functional analysis of a novel nonsense variant c.91A>T of the TRAPPC2 gene in a Chinese family with X-linked recessive autosomal spondyloepiphyseal dysplasia tarda. Front. Genet. 14, 1216592. 10.3389/fgene.2023.1216592 37693308 PMC10492639

[B10] Miyoshi K. Nakamura K. Haga N. Mikami Y. (2004). Surgical treatment for atlantoaxial subluxation with myelopathy in spondyloepiphyseal dysplasia congenita. Spine (Phila Pa 1976) 29 (21), E488–E491. 10.1097/01.brs.0000143621.37688.f3 15507788

[B11] Richards S. Aziz N. Bale S. Bick D. Das S. Gastier-Foster J. (2015). Standards and guidelines for the interpretation of sequence variants: a joint consensus recommendation of the American College of medical genetics and Genomics and the Association for Molecular pathology. Genet. Med. 17 (5), 405–424. 10.1038/gim.2015.30 25741868 PMC4544753

[B12] Tiller G. E. Hannig V. L. Dozier D. Carrel L. Trevarthen K. C. Wilcox W. R. (2001). A recurrent RNA-splicing mutation in the SEDL gene causes X-linked spondyloepiphyseal dysplasia tarda. Am. J. Hum. Genet. 68 (6), 1398–1407. 10.1086/320594 11326333 PMC1226126

[B13] Wang H. Wu W. Xu Z. Xie J. (2013). A novel splicing mutation in the SEDL gene causes spondyloepiphyseal dysplasia tarda in a large Chinese pedigree. Clin. Chim. Acta 425, 30–33. 10.1016/j.cca.2013.07.002 23876379

[B14] Whyte M. P. Gottesman G. S. Eddy M. C. McAlister W. H. (1999). X-linked recessive spondyloepiphyseal dysplasia tarda. Clinical and radiographic evolution in a 6-generation kindred and review of the literature. Med. Baltim. 78 (1), 9–25. 10.1097/00005792-199901000-00002 9990351

[B15] Won J. Y. Kim D. Park S. Y. Lee H. R. Lim J. S. Park J. H. (2019). Novel loss-of-function variants of TRAPPC2 manifesting X-linked spondyloepiphyseal dysplasia tarda: report of two cases. BMC Med. Genet. 20 (1), 70. 10.1186/s12881-019-0802-2 31053099 PMC6500034

[B16] Xiaozhen S. Fan Y. Fang Y. Xiaoping L. Jia J. Wuhen X. (2021). Novel truncating and missense variants in SEMA6B in patients with early-onset epilepsy. Front. Cell Dev. Biol. 9, 633819. 10.3389/fcell.2021.633819 34017830 PMC8129541

[B17] Zappa F. Intartaglia D. Guarino A. M. De Cegli R. Wilson C. Salierno F. G. (2024). Role of trafficking protein particle complex 2 in medaka development. Traffic 25 (1), e12924. 10.1111/tra.12924 37963679

[B18] Zhang C. Du C. Ye J. Ye F. Wang R. Luo X. (2020). A novel deletion variant in TRAPPC2 causes spondyloepiphyseal dysplasia tarda in a five-generation Chinese family. BMC Med. Genet. 21 (1), 117. 10.1186/s12881-020-01052-8 32471379 PMC7260818

