# Supplementary material for: Identification and functional analysis of a novel TRAPPC2 intronic variant in a four-generation Chinese pedigree with SEDT
Source: Front Genet. 2026 Feb 9;17:1763609. doi: 10.3389/fgene.2026.1763609 (PMC12925630; doi:10.3389/fgene.2026.1763609)
Supplement: Supplementary file 1 [file Table1.docx]

**Supplementary Material**

**Identification and functional analysis of a novel *TRAPPC2* intronic variant in a four-generation Chinese Pedigree with SEDT**

Yongfen Lyu, Wuhen Xu, Bin Xu, Xiaojun Tang, Man Xiao, Xiaoping Lan, Yongchen Yang, Xiaozhen Song, Shengnan Wu

Table S1. Primers used in minigene assay

| Primer | Primer sequence |
| --- | --- |
| 2468-TRAPPC2-F | gtgctaggattacaagcata |
| 2778-TRAPPC2-F | agcgccatttcaactgtctc |
| 4023-TRAPPC2-R | agtttcctgacaaaggtcga |
| 4270-TRAPPC2-R | ataggctgctttcttgctgt |
| pcMINI-C-TRAPPC2-KpnI-F | ggtaGGTACCggctgtttctgttgagatgt |
| pcMINI-C-TRAPPC2-BamHI-R | TAGTGGATCCATGCCCCGCAGTGACAAATGCC |
| TRAPPC2-MUT-F | tgtggttgctcacaGtgtcatctagGACGA |
| TRAPPC2-MUT-R | TCGTCctagatgacaCtgtgagcaaccaca |

Table S2. Primers used in *TRAPPC2* expression analysis

|  | Primer | Primer sequence |
| --- | --- | --- |
| Constructions | phage-TRAPPC2-SalI-F | TGACGTCGACcATGTCTGGGAGCTTCTACTT |
|  | phage-TRAPPC2-NotI-R | CGACGCGGCCGCcGCTTAAAAGGTGTTTCTTCC |
|  | GFP-TRAPPC2-HindⅢ-F | GCTCAAGCTTctATGTCTGGGAGCTTCTACTT |
|  | GFP-TRAPPC2-BamHI-R | CGGTGGATCCTCAGCTTAAAAGGTGTTTCT |
| RT-PCR | TRAPPC2-EGFP-QPCR-F | CGACAACCACTACCTGAGCA |
|  | TRAPPC2-EGFP-QPCR-R | TGGTGGCCAACAATTACAAA |
|  | TRAPPC2-phage-QPCR-F | ATCCACGCTGTTTTGACCTC |
|  | TRAPPC2-phage-QPCR-R | TGGTGGCCAACAATTACAAA |
